# Increasing Value of Winery Residues through Integrated Biorefinery Processes: A Review

**DOI:** 10.3390/molecules27154709

**Published:** 2022-07-23

**Authors:** Rafaela P. Rodrigues, Licínio M. Gando-Ferreira, Margarida J. Quina

**Affiliations:** CIEPQPF, Department of Chemical Engineering, University of Coimbra, Rua Sílvio Lima, Pólo II–Pinhal de Marrocos, 3030-790 Coimbra, Portugal; lferreira@eq.uc.pt (L.M.G.-F.); guida@eq.uc.pt (M.J.Q.)

**Keywords:** winery residues, integrated biorefinery, value-added products, energy, circular economy, grape pomace, wastewater

## Abstract

The wine industry is one of the most relevant socio-economic activities in Europe. However, this industry represents a growing problem with negative effects on the environment since it produces large quantities of residues that need appropriate valorization or management. From the perspective of biorefinery and circular economy, the winery residues show high potential to be used for the formulation of new products. Due to the substantial quantities of phenolic compounds, flavonoids, and anthocyanins with high antioxidant potential in their matrix, these residues can be exploited by extracting bioactive compounds before using the remaining biomass for energy purposes or for producing fertilizers. Currently, there is an emphasis on the use of new and greener technologies in order to recover bioactive molecules from solid and liquid winery residues. Once the bio compounds are recovered, the remaining residues can be used for the production of energy through bioprocesses (biogas, bioethanol, bio-oil), thermal processes (pyrolysis, gasification combustion), or biofertilizers (compost), according to the biorefinery concept. This review mainly focuses on the discussion of the feasibility of the application of the biorefinery concept for winery residues. The transition from the lab-scale to the industrial-scale of the different technologies is still lacking and urgent in this sector.

## 1. Introduction

Nowadays, the increasing environmental pollution and the exploitation and depletion of natural resources have become some of the most concerning subjects in the scientific community. The agri-food industries have been responsible for the high consumption of natural resources, particularly water, and at the same time for the production of large amounts of residues (solid or liquid). According to Ahmad et al. (2020), the wine industry is among the biggest contributors to agricultural waste accumulation [1]. The wine industry is one of the most important socio-economic activities in the world, particularly in Europe. According to the 2019 statistical report on world vitiviniculture, in 2018 there was a worldwide production of 292 million hL of wine, 65% of which was produced in Europe [2]. However, the wine industries require large amounts of water (1–4 L per liter of wine produced, globally resulting in 292–1168 million hL of water consumption) and generate a substantial amount of solid and liquid residues throughout the winemaking process [3,4]. According to Oliveira and Duarte (2016), per 1000 kg of grapes processed there is the production of 750 L of wine (desired product) but also the generation of 1650 L of wastewater and around 200 kg of solid residues [5]. Traditionally, the solid fractions have been directly disposed of into large open fields, whereas winery wastewater has been treated in aeration tanks [5]. This makes winery waste a source of environmental pollution due to the discharge of high organic loads associated with volatile organic compounds [1,6,7]. On the other hand, following sustainable approaches, winery residues can be exploited in order to become a source of value-added products [8,9,10]. According to Teixeira et al. (2014), these residues contain a large amount of secondary metabolites, namely, phenolic compounds, including phenolic acids (hydroxybenzoic and hydroxycinnamic acids), flavonoids (mainly flavanols, flavonols, proanthocyanidins, and anthocyanins), and stilbenes [11]. According to the available studies, the main biological functions of these compounds are the antioxidant, antimicrobial, anti-inflammatory, and anticancer activities [11,12,13]. In fact, the antioxidant activity has been one of the most attractive characteristics of phenolic compounds [11]. The radical scavenging activities of these metabolites are supported by their reducing power through hydrogen donating and single oxygen quenching [11]. These activities allow these compounds to interact with biological systems, preventing, for example, degenerative diseases linked to oxidative stress [11]. Therefore, winery residues have become an attractive source of food additives, dietary supplements, cosmetics, dyes, biofuels, and fertilizer, among many other products that can be generated from these residues [6,14,15,16,17,18]. Recent studies demonstrated the potential of winery solid residues for the recovery of natural antioxidant compounds (polyphenols), tartaric acid, bio-oil, fertilizers, biosurfactants, and biogas, among others [6,14,15,18]. Several value-added compounds have been recovered from winery wastewater through membrane processes [19,20]. Additionally, after depuration, wastewater has a great potential to be a renewable source of irrigation water, allowing for the reduction in freshwater consumption. 

Although the valorization of individual winery waste streams has been explored, there is a need to shift from linear practices to sustainable and circular approaches [21,22]. The biorefinery approach can be defined as the integration of various processes that aim to produce a variety of valuable products (energy, green chemicals, and material compounds) from biomass and waste, allowing for the ultimate cascade valorization of these substrates. Indeed, biorefinery has been recognized as a remarkable solution for the valorization of waste, which should be implemented more and more [23,24,25]. Furthermore, from an ideal point of view, the integrated biorefinery concept aims to achieve “zero waste” production, according to a circular economy framework, using residual waste as a raw material [23].

This review aims to promote the discussion about the feasibility of the application of the integrated biorefinery concept for winery residues. Moreover, the transition from the lab-scale to industrial-scale of the different technologies is also analyzed by providing the state-of-art knowledge on the current developments.

## 2. Winemaking Process

**Figure 1:**
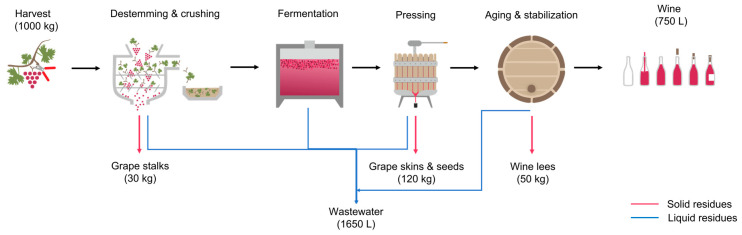
Schematic representation of red winemaking process.

Wine production is a seasonal process that occurs between August and October in the northern hemisphere and between January and April in the southern hemisphere [4]. As can be seen in Figure 1, the grapes are initially harvested, destemmed (stalks removal) and semi-crushed. In the production of red wine, the crushed grapes and the juices (must) are then transferred to an open container where the sugar fermentation into alcohol is initiated by yeasts. 

After the fermentation step, the must is mechanically pressed, and the juice is separated from the grape skins, seeds, and remaining stalks (grape pomace). The juice is then transferred into closed vessels to begin the aging and stabilization process. In this step, the yeast and any remaining grape solids are sedimented at the bottom of the container (wine lees). The bottling occurs when the wine is mature enough. For the production of white wine, all the steps described are similar, except for the pressing step, which takes place before the fermentation step [26]. 

During winemaking, there is a generation of huge amounts of residues, solid and liquid, from each process stage. Regarding the solid residues, according to Oliveira and Duarte (2016), during the destemming phase, there is a generation of 30 kg of stalks and 120 kg of grape skins and seeds (grape marc) per 1000 kg of grapes harvested [5]. In some processes, there is no destemming step, and a combined residue of stalks, seeds, and skins (grape pomace) is generated. 

After the aging and stabilization process, there is the formation of wine lees, which are usually mixed with the wastewater from the different washing operations throughout the entire process [1]. 

## 3. Winery Waste Composition

### 3.1. Winery Wastewater

The winery wastewater is characterized by an acidic pH, phytotoxic, and with a high organic load (chemical oxygen demand—COD up to 296 g L^−1^), as can be seen in Table 1. According to Ioannou et al. (2015), the high organic load is due to the presence of carbohydrates, lipids, proteins, and phenolic compounds [27]. Without proper treatment, through aerobic processes (aeration), the discharge of the wastewater can result in a very negative impact, namely, the death of aquatic organisms and the generations of bad odors as a result of a reduction in oxygen levels in the aquatic media due to high COD values [5,28]. The treatment of wastewater poses specific difficulties because, in addition to the high organic/inorganic load, the treatments have to manage large volumes with considerable seasonal variability. The treatments may involve physicochemical, biological, membrane filtration and separation, advanced oxidation processes, and combined biological and advanced oxidation processes [27]. 

The combination of physicochemical and biological processes is the most commonly applied strategy on the industrial scale. According to Iaonnou et al. (2015), the physicochemical processes, such as coagulation/flocculation and electrocoagulation, have been used as a pre-treatment of winery wastewaters due to the capacity of these processes to lower the COD and total solid content [27]. Moreover, the biological processes, aerobic and anaerobic, allow for the treatment of these wastewaters since the majority of the organic load, after the pre-treatment, is easily biodegradable [27]. 

### 3.2. Grape Seeds

According to the results shown in Table 2, grape seeds are mainly composed of carbohydrates (64.7–72.1%, dry basis), proteins (8.8–12.6%, dry basis), and lipids (7.2–24.8%, dry basis). 

According to Fernandes et al. (2013), the lipid content of grape seeds is mainly composed of linoleic acid (up to 73.1%), followed by oleic acid, palmitic acid, and stearic acid [41]. The grape seeds show great potential for nutraceutical applications [42]. In fact, grape seed extracts can be used in the treatment of human diseases, as dietary supplements, as well as in the formulation of cosmetics [42,43,44] According to Lin et al. (2014) and Salehi et al. (2019), it is possible to recover gallic acid, hydroxybenzoic, and cinnamic acid derivatives, as well as other similar compounds through extraction processes, namely, through solid–liquid and high-pressure-and-temperature extraction using methanol as solvent [45,46,47,48]. The bioactive compounds that can be extracted from grape seeds exhibited good antioxidant properties due to the presence of natural phenolic compounds [49].

### 3.3. Grape Stalks

According to Table 3, grape stalks are rich in dietary fibers (up to 77% of dry weight), which are mainly composed of lignin (4.6–47.3%, dry basis) and structural carbohydrates (cellulose and hemicelluloses) [35]. Moreover, Llobera and Cañellas (2007) and Karvela et al. (2009) highlighted that grape stems can have the potential to be a source of proanthocyanidins, flavan-3-ols, hydroxycinnamic acids, stilbenes, monomeric and oligomeric flavonols [50,51]. According to Troiolo et al. (2021), the stilbenes present in grape stalks can be added to the wine and act as substitutes of SO_2_ [52]. The stilbenes present in grape stalks can be added to the wine and act as substitutes for SO_2_. Moreover, the addition of stilbenes to the wine can improve the intensity, purity, color and increase the storage time in the bottle. Functionalized grape stalk powder can also be used as a reinforcing filler in polybutylene succinate, usually used for packing and agriculture, allowing for the reduction in polymer consumption [53]. Moreover, grape stalks can be used as a bulking agent for composting processes [1].

### 3.4. Grape Skins

Regarding grape skins, the characterization reported in the literature is scarce. According to Romo Sánchez et al. (2015), the skins are mainly composed of lignocellulosic compounds such as cellulose, hemicelluloses, and lignin, as can be seen in Table 4 [56].

Gerardi et al. (2021) and Baron et al. (2021) reported that grape skin extracts showed high antioxidant and antibacterial activity. Moreover, red grape skin extracts were found to have better properties than those obtained for white grape skin extracts [57,59]. Due to the properties observed, the obtained extracts can be used as natural preservatives in food industries and in pharmaceutical/cosmetic sectors as additives [57,59].

### 3.5. Grape Pomace

The grape pomace (GP) is the main solid residue generated in the winemaking process, representing between 10 and 30% *w*/*w* of the initial feedstock [11,60,61,62]. The general composition of grape pomace are summarized in Table 5. According to Hogervorst et al. (2017), the main fraction of GP is grape skins (47%), followed by the stalks (28%) and seeds (25%) [63]. As can be seen in Table 5, GP contains high quantities of lignin (32.5–56.7%) due to the presence of stalks and skins, and high lipid content due to the presence of seeds. The grape pomace is well known for the presence of phenolic compounds, mainly in grape skins, which can be recovered and used for nutraceuticals, medical remedies, and also applied in the cosmetic and food industry [64]. Several studies have exhibited the suitability of GP as a source of natural antioxidants and phenolic compounds [48], whereas the cellulose and hemicellulose present in GP can be used to produce biofuels through fermentation and anaerobic digestion processes [1]. 

## 4. Winery Residues Biorefinery 

The integrated biorefinery approach allows for the possibility to obtain bio-based products (biochemicals, energy, heat, or biofuels), applying different processes in cascade. This strategy may have a significant positive impact on the revenue and product diversification of industries [1]. The conversion of biowaste into high-value products and energy through biorefinery is a very desirable strategy that should be implemented in the near future, not only because of environmental protection but also for economic reasons [69].

**Figure 2:**
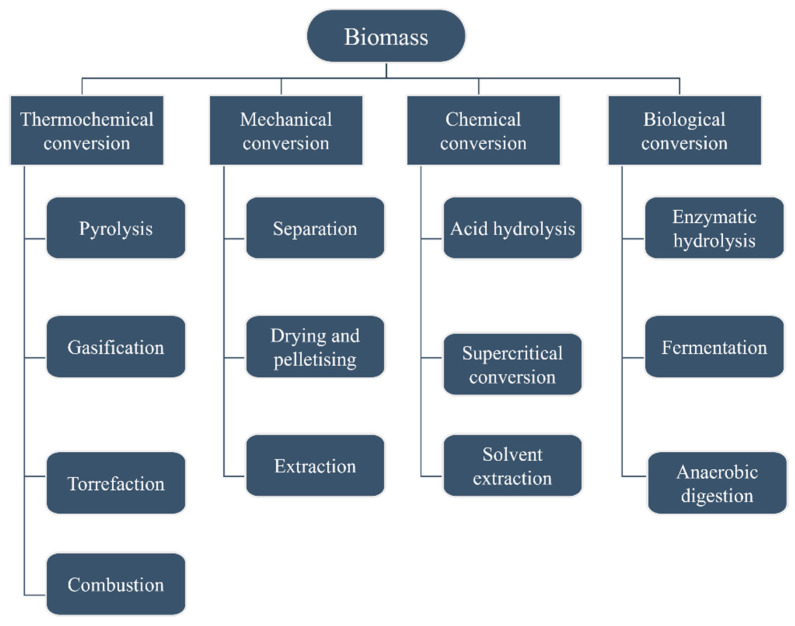
Schematic structure of biomass conversion processes (based on [71]).

According to Velvizhi et al. (2022), biorefineries can be classified as energy-driven or product-driven based on the selected biorefinery pathways [70]. As can be seen in Figure 2, biorefinery may integrate four main process types: mechanical conversion, chemical conversion, biological conversion, and thermochemical conversion [71].

According to Ahmad (2020), the solid and liquid fractions of winery residues have been used successfully in the biorefinery context [1]. In general, solid fractions can be used for conventional biorefinery processes (such as extraction, pyrolysis, gasification, anaerobic digestion, fermentation, among others), whereas wastewater can be valorized through bioconversion processes (essentially anaerobic digestion and fermentation). However, in practice, only a few studies of integrated valorization of winery waste were found in the literature. The current studies are more focused on the feasibility of winery waste in single processes than in cascade approaches. The main focus of these studies is the recovery of bioactive compounds, energy production, and compost/fertilizer production.

### 4.1. Recovery of Value-Added Products from Winery Residues

The residues generated from the wine industry contain a large number of valuable bioactive phytochemicals [60]. The phytochemicals (bioactive nutrient plant chemicals) present in grapes are known to have anticancer, antioxidant, anti-inflammatory, antimicrobial as well as cardioprotective and neuroprotective properties [72,73]. According to Rani et al. (2020), the phytochemicals can be classified into five main categories: carotenoids, alkaloids, phenolic compounds, organosulphur compounds, and nitrogen-containing compounds [60]. Grape phytochemicals, for example, have been identified essentially as rich materials in carotenoids and phenolics, the latter being in a higher quantity [73].

Phenolic compounds are one of the largest groups of phytochemicals and have become an attractive source of natural antioxidants associated with several health benefits mainly due to their strong antioxidant activity [60]. Phenolics can mainly be divided into phenolic acids, flavonoids and stilbenes [11]. The phenolic acids can be sub-divided into two main groups: the hydroxybenzoic and hydroxyxcinnamic acids. In winery residues, the hydroxybenzoic acid derivatives are mainly represented by gallic acid, p-hydroxybenzoic acid, protocatechic acid, syringic acid, tannic acid and vanillic acid. The first one is described as the most abundant hydroxybenzoic acid derivative in grape stalks and seeds [12,74,75,76], whereas protocatechuic acid is most abundant in grape skins and pomace [77,78]. According to Teixeira et al. (2014), the differences observed in the phenolic profile of grape pomace and its individual constituents may be due to the presence of traces of grape pulp in the pomace, which can increase phenolic content [11]. The hydroxycinnamic acids can be detected in all individual parts of grapes, where the highest concentration can be found in grape skins. The flavonoids are low-molecular-weight compounds that can be sub-classified as flavonols, flavanols, flavones and anthocyanins [11]. The distribution of flavonols in the individual winery residues present some differences regarding the different flavonols as well as to their concentration. In general, grape stems is the fraction that present the higher variety of flavonols, with high contents of quercetin derivatives (quercetin-3-O-glucuronide, quercetin-3-O-glucoside, quercetin-3-O-galactoside, and quercetin-3-Orutinoside) [12,50,79,80]. The grape pomace and grape skins also present high concentrations of quercetin-3-O-glucuronide and quercetin-3-O-glucoside [65,76,81,82]. According to Teixeira et al. (2014), the lack of data on the grape seed content in flavonols can indicate that these compounds have no significant importance in grape seeds [11]. The flavanol concentrations in stems are higher than those observed in grape skins and seeds, and catechin is the main flavanol observed in both stems and skins. Tannins are a more complex and higher molecular weight class of phenolic compounds [11]. These compounds can be divided into two groups: condensed tannins and hydrolysable tannin. Condensed tannins, also known as proanthocyanidins, are constituted by subunits of flavanol monomers, whereas hydrolyzable tannins are complex polyphenolics that can be degraded into smaller units such as sugars and phenolic acids [11]. Condensed tannins (procyanidin dimers B1, B2, B3, and B4 and procyanidin trimer C1) represent a significant proportion of these residue phytochemicals, even though the concentrations in the grape stems are more significant than the one observed for seeds and skins. Anthocyanins are highly soluble flavonoids that are directly responsible for red color grapes and wines ([11]. Naturally, the anthocyanins are present in the grape skin and, as a consequence, in grape pomace. The anthocyanins consist of an aromatic ring (aglycone anthocyanidin) bonded to an oxygen-containing heterocyclic ring, which is also bonded to a third aromatic ring. The third aromatic ring forms conjugates with sugars and organic acids generating a variety of anthocyanins that have distinct associated colors [11]. Stilbenes (trans-resveratrol and ε-viniferin) are phenolic compounds that are mainly found in grape stems. These compounds are produced as a response to physiological stressing factors, such as ozone and UV-C radiation [11]. These compounds can be recovered from grape pomace mainly by extraction processes, whereas membrane processes are used in the winery wastewater. To date, there is no standard extraction method for the recovery and identification of compounds, namely, phenolic molecules in solid matrixes [83]. 

#### 4.1.1. Solid–Liquid Extraction

Despite this, the most common and traditional extraction techniques for the recovery of phenolic compounds are solid−liquid extractions [84]. Some of the published studies regarding the solid–liquid extraction (SLE) of winery residues and the obtained phenolic content (TPh) are summarized in Table 6. 

SLE is based on mass transport phenomena, mainly affected by the extraction solvent type, temperature, and extraction time. The solvent choice is one of the most critical operational parameters, due to the polar nature of polyphenols. In general, the best extraction efficiencies can be obtained by using polar protic media such as hydroalcoholic solutions [83]. As can be observed in Table 6, the most common solvents used for the extraction of phenolic compounds are methanol, ethanol, acetone, and water. According to Yilmaz and Toledo (2006), as pure solvents, methanol exhibits a higher extraction capacity, followed by ethanol, acetone, and water [97]. However, several studies concluded that the addition of water as a co-solvent increases the phenolic extraction when compared to pure solvents [85,87,95]. In fact, Yilmaz and Toledo (2006) reported a total phenolic content three times higher when using a dilution of 50% instead of pure acetone as solvent [97]. However, the published results are not consensual about the ideal solvent for the extraction of polyphenols from winery residues, since the different combinations of the operational conditions can produce similar results. The extraction time and temperature are also among the most important parameters to be optimized in order to achieve high recoveries of compounds. According to the published results, an increase in the working time enhances the extraction efficiency until a certain point [85,91]. The increase in the extraction temperature also improves the phenolic extraction [85,85,91]. However, according to Fontana et al. (2013), this factor cannot be increased indefinitely since the phenolic compounds become unstable at temperatures above 50 °C [83]. Makris et al. (2007) and Negro et al. (2003) studied the different components of grape pomace (stalks, seeds, and skins) [88,90]. The reported outcomes indicate that the extraction of grape seeds resulted in higher phenolic content followed by grape stalks, pomace, and skins. Moreover, the extracts of grape seeds exhibited higher antioxidant activity due to the presence of higher content of flavonoids, namely, proanthocyanins [88,90]. Rockenbach et al. (2011) studied four varieties of grape pomace and concluded that the total phenolic content extracted depends on the pomace origin, and the results showed that the TPh ranged from 32.62 to 74.75 mg_GAE_ g_db_^−1^ [48]. Although SLE has been used for many decades, these processes are generally time-consuming and use large quantities of organic solvents [85]. Moreover, in order to establish more environmentally friendly extraction methods, the use of new green alternative solvents, such as natural deep eutectic solvents (NADES), has been explored. The NADES are liquid salts that are generally mixed with naturally derived uncharged hydrogen-bond donors that provide the green profile to the solvent [96]. Bosiljkov et al. (2017) studied the SLE extraction of wine lees using different NADES and compared the results obtained with the extracts obtained with ethanol:water:formic acid (50:48.5:1.5 %) [96]. The results indicated that the use of choline chloride:malic acid (1:1) mixed with water (25%) allowed for higher extraction of anthocyanins (5.5 mg anthocyanins g^−1^, db) compared to the results obtained with conventional solvent (about 4.4 mg anthocyanins g^−1^, db). The results suggest that conventional solvents can be replaced with new greener alternatives without compromising the overall extraction performance.

#### 4.1.2. Ultrasound-Assisted Extraction

Recently, some new techniques have been studied, with a focus on the extraction time reduction as well as on the reduction in the organic solvent consumption without compromising the recovery of phenolic compounds [85]. In this context, the application of ultrasounds has been explored as an alternative to conventional SLE. Some of the published results regarding the application of ultrasound-assisted extraction (UAE) in the phenolic compounds recovery from winery residues are summarized in Table 7. Caldas et al. (2018), Da Rocha and Noreña (2020), and Grillo et al. (2020) compared the UAE with the conventional SLE [86,91,93]. At the same operational conditions (solvent, temperature, and time), for example, Grillo et al. (2020) concluded that the use of ultrasound technology allowed for an increase from 27.09 to 31.89 mg_GAE_ g_db_^−1^ in the extraction of phenolic content [93]. Moreover, Da Rocha and Noreña (2020) observed a higher phenolic extraction (from 3 to 4.5 mg_GAE_ g_db_^−1^) but with a considerable extraction time reduction (from 24 h to 15 min) when using UAE instead of SLE [91]. The efficiency of UAE extraction might be justified by the enhancement of the hydration and fragmentation process, along with the increase in the mass transfer capacity of solutes into the solvent [83].

The increase in the ultrasound frequency has been reported to increase the extraction of phenolics [91,98,102]. This effect might be due to the improved cavitation, which increases the contact surface area between solid and liquid surfaces, allowing for greater penetration of the solvent into the solid matrix [98]. However, Drevelegka and Goula (2020) observed that above certain ultrasound amplitudes the extraction performance decreased possibly due to degradation of the material [98]. 

#### 4.1.3. Microwave-Assisted extraction

Another non-conventional technology is microwave-assisted extraction (MAE). This extraction process is based on the use of microwaves (300 MHz–300 GHz). This type of electromagnetic radiation is able to heat up the molecules of polar solvents due to their dual mechanism of ionic conduction and dipole rotation [106]. The ionic conduction occurs by the migration of ions due to the electric field changes, which generates a solution resistance resulting in friction and consequently the solution gets heated up [106,107,108]. The generated localized heat will cause the evaporation of the water molecules present in the sample, causing a build-up pressure that leads to the cell matrix rupture and consequently release/leaching of the active compounds [108]. The summary of the published results obtained with this method is presented in Table 8.

When comparing the performance of MAE and SLE, Da Rocha and Noreña (2020) observed that the use of microwave radiation allowed for an increase in the phenolic extraction from 3 to 7 mg_GAE_ g_db_^−1^ in a shorter extraction time (10 min instead of 24 h) [91]. Additionally, MAE was revealed to have a better performance when compared to UAE [91,98]. MAE, like UAE, exhibited higher extraction efficiencies with higher working power. 

#### 4.1.4. Accelerated Solvent Extraction

Accelerated solvent extraction (ASE), also known as pressurized solvent extraction, is an alternative solid liquid extraction technique that allows for the use of common solvents used in other extraction techniques, but at subcritical conditions, i.e., increased pressure (100–140 bar) and elevated temperatures (50–200 °C) [106]. 

The high pressure allows for the extraction temperature to be raised above the boiling point of the solvent while maintaining the solvent in a liquid state. At high temperatures, the solvent has some properties such as high diffusion coefficients, low viscosity and high solvent strength that increase the extraction process efficiency and kinetics [83,106]. Moreover, the high pressure allows the solvent to penetrate deeper into the sample matrix, increasing the extraction of analytes. Moreover, the amount of solvent and extraction time can be reduced by ASE instead of SLE. In fact, Aliakbarian et al. (2012) observed an enhancement in the TPh extracted from 7.87 to 32.49 mg_GAE_ g_db_^−1^ in a shorter time (2 h) when using ASE instead of SLE, as can be seen in Table 9 [92].

#### 4.1.5. Supercritical Fluid Extraction

Supercritical fluid extraction (SFE) is a technique that aims to extract target analytes from solid matrices using supercritical fluids [83]. The published studies regarding SFE application for the recovery of phenolic compounds from winery residues are summarized in Table 10. 

As can be seen in Table 10, CO_2_ is the most common solvent used in SFE. Due to the low polarity of carbon dioxide, several studies indicate the benefit of the incorporation of polar solvents, such as ethanol, in order to enhance the extraction of polyphenols [113,114,115]. De Campos et al. (2008) observed an increase from 3 to 9% in the extraction yield when 15% of ethanol was used as co-solvent instead of pure CO_2_ [116].

#### 4.1.6. High-Voltage Electrical Discharges Extraction

Recently, the use of high-voltage electrical discharges (HVED) has become an interesting process to extract bioactive compounds from solid matrixes. HVED is an extraction technique based on the application of a high voltage between two submerged electrodes [83]. Upon the application of the electric field, the electrons are accelerated and reach sufficient energy in order to excite water molecules and a stream of electrons is created [117]. This electron stream (streamers) will propagate from the positive to the negative electrode and, when the streamers reach the negative electrode, an electrical breakdown occurs. According to Brianceau et al. (2016), such a phenomenon will create high-amplitude pressure shock waves, bubble cavitation, and liquid turbulence [118]. This phenomenon, as in UAE, will cause particle fragmentation and cell structure damage, which will accelerate and improve the extraction process [117,118]. Table 11 highlights some of the published works regarding winery residues applying this technique. Boussetta et al. (2011) obtained 10 times more phenolic compounds when HVED was applied instead of SLE [119]. Brianceau et al. (2016) studied the impact of pH, treatment time and ethanol concentration in the extraction of phenolic compounds, in particular the recovery of flavan-3-ols, flavonols and stilbenes, through HVDE [118]. The results demonstrated that the pH has a significant impact on the recovery of these compounds. The recovery of flavonols increased with higher pH levels, whereas the extraction of flavan-3-ols and stilbenes was negatively affected by the pH increase.

The treatment time was found to be important for the extraction of flavan-3-ols and flavonols, but no significant impact was found on stilbenes extraction. The most important parameter, similar to other extraction processes, was found to be the ethanol concentration for the three individual compounds. After optimization, the best conditions were found to be a pH of 2.5, a treatment time of 4 ms and a 50% ethanol solvent. Moreover, when compared to the SLE, the application of HVED allowed for an increase of 35% in the total phenolic compounds, 32% in flavan-3-ols and 12% in flavonols recovered. Barba et al. (2015) compared the performance of UAE and HVED techniques in the extraction of phenolic compounds [121]. Overall, HVED allowed for the extraction of two times the amount of compounds recovered by UAE. Moreover, the application of electrical discharges revealed to be a less energy consuming process for the same quantity of phenolic compounds extracted. However, the results indicated a low selectivity of the HVED process when it comes to the extraction of anthocyanins. Therefore, this process must be optimized. A scale-up study conducted by Boussetta et al. (2012) suggested that higher energy per pulse is needed for pilot-scale HVED extraction processes in order to obtain similar results to those obtained on a laboratory scale [118].

Overall, according to the studies available, the winery residues are a good source of phenolic compounds and antioxidants. However, it is not possible to conclude which process is the best for the extraction of phenolic compounds, and all processes must be optimized for each different matrix (pomace, seeds, stalks, and seeds). Table 12 summarizes the main operating conditions of each extraction process.

Globally, it is possible to highlight that the extraction time of phenolic compounds decreases with non-conventional extraction methods when compared with the SLE extraction. Moreover, UAE and MAE methods can be a good alternative since a shorter extraction time is needed. HVED in UAE and MAE tend to be more based in hydroalcoholic mixtures relatively to SLE which is a bonus when it comes to the application of the extracts in food and cosmetic industries as well as moving towards eco-friendly processes. However, other factors such as product safety and general cost have a huge impact on the extraction method selection [83]. 

Most of the non-conventional methods have been tested only on a laboratory scale, so pilot-scale studies are necessary to select the optimal technique after evaluating the costs to produce commercial reliable extracts.

Altogether, when comparing the phenolic compound extraction results and the main advantages/disadvantages of the mention extraction processes, the UAE seems the most promising extraction process due to the reduced operational time and high phenolic concentration extraction obtained

#### 4.1.7. Membrane Processes

According to Teixeira et al. (2014), the phenolic compounds recovered from winery residues can be classified as phenolic acids, flavonoids, and stilbenes [11]. The relative proportion of the polyphenols in winery residues has been determined by high-performance liquid chromatography (HPLC). The concentration of the main phenolic compounds in the extracts can be observed in Table 13.

It is important to mention that the recovery of phenolic compounds from winery wastewater is mainly accomplished through membrane processes, namely, ultra and nanofiltration. These membrane processes are a combination of both mass and momentum transfer where a thin barrier (membrane) is placed between two mediums allowing one or more constituents to selectively pass from one medium to the other by means of a driving force [123]. The components present in the feed that are able to pass through the membrane are designated as permeates, whereas the remaining components are known as retentates. Giacobbo et al. (2013a) studied five membranes with different characteristics to evaluate the optimal conditions for the fractionation between polyphenols and polysaccharides [124]. The results demonstrate that the nanofiltration can retain polysaccharides (RTPs > 70%). The membranes with lower cut-off (CA 400-22, CA 400-26, and NF 270) show high retention coefficients for polyphenols (>63%) and polysaccharides (99%). However, a low fractionation between the two compounds is observed. Moreover, the fractionation between the two solutes is achieved through membranes with a higher molecular weight cutoff (MWCO), where the polysaccharides are retained, and the phenolic compounds pass into the permeate. Similar phenolic rejection coefficients with NF 270 and ETNA01PP membranes were obtained by Giacobbo et al. (2018) [125]. Indeed, Giacobbo et al. (2013b) investigated the effect of the pH on the rejection of phenolic compounds and polysaccharides through the ultrafiltration process; the results showed higher phenolic retention at acidic pH and a higher degree of separation of the two solutes at pH 5.4 [28]. Galanakis et al. (2013) obtained similar results, regarding the retention of phenolic compounds [126]. Rodrigues et al. (2020) studied the optimization of the micellar enhanced ultrafiltration (MEUF) for the fractionation of phenolic compounds and polysaccharides using model solutions, where pH and surfactant concentration were found to be key operational parameters [127].

**Table 13:** Phenolic compounds in the extracts from winery residues.

Phenolic Group	Compound	Value (mg g _db_ ^−1^)
Phenolic acids	Gallic acid	0.05–2.45
	Protocatechuic acid	0.01–1.66
	p-Coumaric acid	0.01–0.02
	Vanillic acid	0.07–0.09
Flavonoids	(+)-Catechin	0.02–15.59
	(−)-Epicatechin	0.03–10.23
	Procyanidin B1	3.81–7.80
	Procyanidin B2	2.88–6.07
	Rutin	0.12–0.41
	Quercitin 3-O-Glucoside	0.09–0.74
	Anthocyanins:	
	Delphinidin-3-glucoside	0.05–0.20
	Cyanidin-3-glucoside	0.01–0.02
	Petudin-3-glucoside	0.14–0.53
	Peonidin-3-glucoside	0.15–0.46
	Malvidin-3-glucoside	0.99–5.94
Stilbenes	Resveratrol	0.01–0.06

db—dry basis. References: [48,86,128,129].

MEUF is a modified ultrafiltration separation technique in which a surfactant-containing solution is filtered with an ultrafiltration process [130]. The surface-active agent (surfactant) alters the properties of a solution by changing its surface tension. When surfactant monomers are in a certain concentration in the solution, there is a formation of micelles. The micelle formation allows for the solubilization of certain organic/inorganic compounds. This solubilization can occurs at the micelle–water interface, between the hydrophilic head groups, in the hydrophilic groups and the first few carbon atoms of the hydrophobic groups and in the inner hydrophobic core of micelle. Due to the size of the micelles, they can be rejected by the membrane [130], allowing for the retention of low molecular solutes such as phenolic compounds. The MEUF process demonstrated to be an interesting method for the fractionation of phenolics and polysaccharides of both model solutions and real wastewater [127]. MEUF has been also applied for other agro-industrial wastewaters. Víctor-Ortega et al. (2017) also observed higher phenolic retention with the use of surfactants; the obtained results showed an increase of 70% in the rejection coefficient for the application of MEUF instead of regular UF [131]. Moreover, the nature of the surfactant has been revealed to be an important operational parameter, since the lower rejection of phenolics was obtained for non-ionic, followed by anionic and cationic surfactants. Overall, MEUF exhibited an enhancement in the phenolics rejection for further valorization.

#### 4.1.8. Enzymatic Hydrolysis

Enzymatic hydrolysis is a common intermediate conversion step for biomass processing, aiming at using enzymes to facilitate the cleavage bonds of macromolecules by the addition of water to produce reducing sugar hydrolysate that can be used as a substrate by fermentative organisms. Hydrolytic enzymes can break down macromolecules (e.g., protein, lipids, carbohydrates, and fats) into the simplest units. For example, a cellulose chain can be broken into glucose molecules. In the literature, some studies have used this approach to extract more values from biomass. For example, Jin et al. (2018), tested a commercial enzyme (Cellic CTec 2, activity 132 FPU mL^−1^) in red grape pomace, followed by an incubation period of 72 h, at 50 °C under agitation. Glucose and xylose yield was calculated to evaluate the efficiency of the hydrolysis [132].

### 4.2. Energy Recovery

#### 4.2.1. Biogas

As indicated in Section 3, the winery residues are known to have a high content of organic matter. Thus, anaerobic digestion may be considered to convert the biodegradable organic matter into biogas (a mixture of CH_4_ and CO_2_), which can be further valorized as electrical energy, heat, or fuel gas. Table 14 highlights some of the published works regarding the anaerobic digestion of winery residues and wastewater.

El Achkar et al. (2016) studied three different substrates (grape pomace, seeds, and pulp) as well as the effect of particle size reduction [36]. The results revealed that the size reduction has a positive impact on the biochemical methane potential (BMP) of all substrates. Moreover, through a Pearson correlation coefficient, El Achkar et al. (2016) concluded that the BMP is strongly affected by the hemicelluloses, soluble compounds, cellulose, and lignin, where the first two have a positive impact while the opposite was observed for the last two [36]. Indeed, lignin, cellulose, hemicelluloses, and soluble compounds content were found to be able to explain 63.5% of the BMP variation, through principal component analysis [133]. This analysis demonstrates that higher BMP is observed for pulp (less lignin and cellulose content) with a BMP of 182 L_CH4_ kg_VS_^−1^, followed by grape pomace (170 L_CH4_ kg_VS_^−1^) and seeds (129 L_CH4_ kg_VS_^−1^) [36]. Da Ros et al. (2016) observed the same behavior between the methane production of grape stalks, and skins and seeds [134]. The grape pomace is constituted by a high content of lignocellulosic compounds, which are not easily degradable. Within the winery residues, the wine lees showed higher BMP when compared to the other residues (between 370 and 876 L_CH4_ kg_VS_^−1^). The higher BMP values can be related to the presence of organic acids and sugars in the lees, which are readily biodegradable compounds [134,135,136]. El Achkar et al. (2018) studied several pre-treatments to increase the biodegradability of grape pomace [136]. The results demonstrated that the treatment type influences methane production. The freezing (−20 °C) of grape pomace followed by the addition of 10% NaOH (*w*/*w*, dry basis) allowed for an increase in the BMP of 68% and de biodegradability from 37 to 63% [136]. In fact, the combination of easily degradable substrates (such as wine lees) with non-easily degradable substrates (grape stalks and pomace) allowed for the development of microorganisms, and their metabolism was increased and remained high for longer when compared to single-substrate AD [135]. According to Ma et al. (2019), the main synergetic effect observed in co-digestion processes is the increase in the hydrolysis rate. Moreover, since the hydrolysis is considered, the rate-limiting step in AD that processes the co-digestion allows one to overcome this limitation, allowing for higher methane productions [137]. Montalvo et al. (2020) and Filho et al. (2018) studied the effect of the co-digestion of several winery residues, and some synergetic effects have been observed [135,138]. Montalvo et al. (2020) reported a BMP value for vine shoots of 150 L_CH4_ kg_VS_^−1^ and 115 L_CH4_ kg_VS_^−1^ for grape pomace, but when combined with wine lees the methane produced increased to 500 L_CH4_ kg_VS_^−1^ for both shoots and pomace [135]. Filho et al. (2018) obtained a BMP of 148 L_CH4_ kg_VS_^−1^ for grape pomace, but by combining multiple winery residues an increase in the methane production (212 L_CH4_ kg_VS_^−1^) was observed [138]. Overall, the published results indicate that it is possible to overcome low methane production from low biodegradable residues (vine shoots, grape pomace, and stalks) by applying co-digestion. Carrillo-Reyes et al. (2019) demonstrated the influence of the inoculum to substrate ratio (ISR) in the anaerobic digestion of winery wastewater [139].

**Table 14:** Summary of the biochemical methane potential (BMP) of winery residues.

Sample	Operational Parameters	BMP	BD (%)	Reference
Pomace	T: 37.5 °C; SIR: 1:3 (g_COD_ g_COD_^−1^)	125 ^a^	35.6	[36]
Pomace		170 ^a^	48.7	
Seeds		52 ^a^	14.7	
Seeds		129 ^a^	36.9	
Pulp		165 ^a^	47.1	
Pulp		182 ^a^	51.9	
Stalks	T: 35 °C; SIR: nd	170 ^b^ 115 ^b^ 876 ^b^ ~500 ^b^ ~500 ^b^ ~750 ^b^	nd	[135]
Pomace			nd	
Wine lees			nd	
Wine lees + wine shoots			nd	
Wine lees + stalks + pomace			nd	
Wine lees + stalks + pomace + shoots + WAS			nd	
Skins + seeds	T: 38 °C; SIR: 0.5	206 ^b^	nd	[140]
Winery wastewater	T:36 °C; SIR: nd	20–200 ^c^	nd	[139]
Pomace	T: 37 °C; SIR: 1:3 (g_COD_ g_COD_^−1^)	130.5–219.4 ^a^	37–63	[136]
Pomace	T: 37 °C; SIR: 0.5	148 ^b^ 838 ^b^ 212 ^b^	nd	[138]
Wine lees			nd	
Pomace (43%) + Stems (34%) + PS (9%) + SS (9%) + Lees (5%)			nd	
Pomace	T: 37 °C; SIR: 1:3 (g_COD_ g_COD_^−1^)	104–242 ^a^	29.7–69.0	[133]
Stalks Skins and seeds Wine lees	T: 55 °C; SIR: nd	133 ^b^ 360 ^b^ 370 ^b^	nd	[134]
			nd	
			nd	

BMP—biochemical methane potential, T—temperature; SIR—substract to inoculum ratio, BD—biodegradability, ^a^ NL kg_CODi_^−1^, ^b^ NL kg_VSi_^−1^, ^c^ L kg_CODi_^−1^, nd—not defined, PS—primary sludge, SS—secondary sludge, WAS—waste activated sludges.

Low ISR causes the acidification of the system, resulting in lower methane yields. Moreover, the biogas generated from winery wastewater presented an energy conversion potential of about 7.15 kJ g_CODi_^−1^ [139].

Overall, the winery residues, namely, grape pomace, skins, stalks, seeds, and wastewater, revealed a potential for the production of energy (100–880 L_CH4_ kg_VS_-1), depending on the residue and the selected pre-treatment, in the form of biogas or methane, through anaerobic processes.

#### 4.2.2. Bioethanol and Bio-Oil

Bioethanol is a product obtained through fermentation using biomass as a substrate and can be used as a substitute for petroleum-derived fuel [1]. According to Castilla-Archilla et al. (2019), biofuels obtained through biomass can be classified according to the feedstock used as first-generation biofuels (raw crops as feedstock), second-generation biofuels (biomass residues as feedstock), and third-generation biofuels (microalgae or cyanobacteria as feedstock) [23]. Bioethanol was essentially classified as first-generation fuel since it was mainly produced from crops [141]. However, due to the increasing demand for food worldwide, some bioresidues have been studied as a substitute feedstock for bioethanol production [1]. According to some studies, residual sugars are remaining in grape skins, which make grape pomace a good feedstock for second-generation bioethanol production [58,142]. Corbin et al. (2015) studied the feasibility of bioethanol production from white and red grape pomaces [68]. Their results demonstrated that it is possible to obtain between 270 and 400 L of bioethanol per ton of grape pomace. However, after applying an acid hydrolysis pretreatment, bioethanol production increased by 48%. Similar results were obtained by Mendes et al. (2013), who indicated a total yield of bioethanol of 250 L per ton of grape pomace [58]. Overall, it can be concluded that grape pomace can be used for the production of bioethanol, allowing for the recovery of energy or other applications (as raw material).

Bio-oil is another common biofuel for crude oil substitution that can be obtained through biomass [142]. The available results in the literature regarding the production of bio-oil from winery residues are summarized in Table 15. 

According to Ramos et al. (2009), grape seeds have some potential for bio-oil production due to the presence of high content of unsaturated fatty acids, namely, linoleic acid (69%), oleic acid (19%), palmitic acid (7%) and stearic acid (4%) [144]. Moreover, Bolonio et al. (2019) and Jin et al. (2018) reported similar compositions of bio-oil obtained from grape seeds extraction regarding the saturated and unsaturated fatty acid content (11.0–11.3% and 88.5–89.0, respectively) [132,143]. According to Haro et al. (2018), the bio-oil obtained from grape seeds exhibited good low-temperature properties and flash point, ester content, viscosity, and acid value similar to vegetable oils [146]. Keiluweit et al. (2010) referred that the oil obtained after crushing grape seeds can be used directly in combustion engines [147]. In fact, Lapuerta et al. (2021) compared the performance of commercial diesel and a mixture of 30% fatty acid ethyl esters (FAEE) obtained from grape seeds with 70% commercial diesel [143]. The results demonstrated that there is a significant potential to substitute conventional diesel due to the lower particle and CO emissions.

#### 4.2.3. Hydrogen

Hydrogen is considered one of the most interesting and clean sources of energy due to its high energy density and heating value [148]. Through theoretical analysis, Nadaleti et al. (2021) evaluated the yield of H_2_ production obtained from the co-digestion of urban solid wastes and winery residues. According to the study, the biogas can be sent to a reformer, where it is converted into H_2_ and CO_2_. This process can produce 1 Nm^3^ of hydrogen from the conversion of 0.348 kg h^−1^ of methane. The analysis revealed that in 2018, in several municipalities of Brazil, there was a production of 37.9 Gg_CH4_ year^−1^, which can be converted into 1.09 × 10^8^ Nm^3^ of H_2_ year^−1^. This amount of hydrogen can generate 2.62 TWh. Year^−1^ of electric energy, which corresponds to the reduction of 355 kton of CO_2_ year^−1^ if hydrogen is used as a substitute for fossil fuels.

Bocanegra et al. (2010), studied the gasification process of wine grape slurry using supercritical water (2500 MPa and 1200 °C) [149]. According to the results, the winery slurry generates a gas composed of H_2_ (21.08%), O_2_ (3.32%), N_2_ (6.87%), CH_4_ (14.57%), CO (1.10%), and CO_2_ (53.03%). The authors concluded that gasification through supercritical water is a feasible and promising method to obtain highly calorific value gases, such as hydrogen, through winery slurry. 

Moreover, the aqueous phase reforming (APR) can be used as a replacement for the gasification process [150]. The APR is a process that converts oxygenated hydrocarbons dissolved in water into H_2_ through a catalytic reaction [150]. The APR results in a gas mainly composed of H_2_, CO, CH_4_, CO_2_ and light alkanes. Although no studies were found where winery wastewaters or residues were submitted to APR process, Zoppi et al. (2022) reported a yield between 91.2 and 789.1 mg of H_2_ per g of C for brewery and fruit juice wastewater. Moreover, lignocellulosic biomass can also be used in this process after appropriate pretreatment such as hydrolysis [150]. According to the authors, hydrogen can be produced through a green low-cost technology, such as an aqueous phase reforming process.

#### 4.2.4. Thermal Processes

There are several technologies to convert biomass into energy through thermal processes such as combustion, gasification, and pyrolysis [151]. According to Caputo et al. (2005) and Iakovou et al. (2010), the thermal processes must be selected according to the available quantity and characteristics of the feedstock [152,153]. Several studies evaluated the thermal conversion of winery residues, as summarized in Table 16. The combustion and gasification processes involve the oxidation of biomass and occur at temperatures between 800 and 1000 °C [153]. However, the combustion only releases heat, whereas the gasification originates combustible gas further used as fuel [151]. 

Pyrolysis is another thermal process that can be applied for winery residues, where the biomass is decomposed into gas, liquid-tar (mainly composed by depolymerized cellulose, hemicellulose and fractions of lignin), and solid (biochar) fractions, in the absence of oxygen and at temperatures of around 400–700 °C [151]. In the case of combustion, the heat released may be used directly in the process or to obtain steam, which is then used to produce electricity via a steam turbine and generator [151]. The combustion of grape marc can generate a gas with a lower heating value (LHV) between 5.7 and 19.8 MJ kg^−1^ [154,155]. Fiori and Florio (2010) simulated through Aspen Plus the combustion of grape marc and obtained a gas with a higher heating value (HHV) of 21.8 MJ kg^−1^ and an electric and thermal efficiency of 23 and 64%, respectively [156]. Moreover, the simulation of the combustion process was compared with two different gasification processes: air gasification and steam gasification. Fiori and Florio (2010) concluded that the results from the three processes were similar, but combustion may be preferred over gasification processes due to the overall simplicity of the process [156].

**Table 16:** Summary of the thermal conversion of winery residues.

Process		T (°C)	By-Products	Reference
Combustion	Grape marc pellets	nd	Gas:	[155]
			LHV: 19.8 MJ kg^−^^1^	
	Grape marc	nd	Gas:	[156]
			LHV: 5.7–7.9 MJ kg^−1^	
	Grape marc	nd	Gas:	[157]
			HHV: 21.8 MJ kg^−1^	
Air gasification	Grape marc	990	Gas:	[157]
			LHV: 4.2 MJ kg^−1^	
	Vine residues	800	Gas:	[158]
			Yield: 36%	
			CO: 17% H2:10% CO2: 11% CH4: 4% C2H6: 11%	
			LHV: 6.6 MJ m^−3^	
Steam gasification	Grape marc	652	Gas:	[157]
			LHV: 9.4 MJ kg^−1^	
Pyrolysis	Grape marc	500	Biochar:	[159]
			Yield: 33%	
			K: 2.2%	
			P: 0.62%	
			N: 2.7 %	
			Fixed carbon: 58.2%	
	Grape marc	500	Biochar:	[160]
			Yield: 38%	
			HHV: 30.2 MJ kg^−1^	
			Tar:	
			Yield: 34%	
			HHV: 30.5 MJ kg^−1^	
	Vine residues	550	Gas:	[161]
			Yield: 43%	
			HHV: 10.6 MJ kg^−1^	
			Tar:	
			Yield: 17%	
			HHV: 5.7 MJ kg^−1^	
			Biochar:	
			Yield: 40%	
			HHV: 12.8 MJ kg^−1^	
			K: 2.3%1	
			P: 0.1%	
			N: 0.5%	
			Fixed carbon: 40.2%	

HHV—higher heating value; LHV—lower heating value; nd—not defined; T—temperature.

A life cycle analysis approach was used to evaluate the overall environmental performance of heat production from grape marc combustion, and the results showed that this residue is a promising feedstock for the production of bioenergy [154].

Pyrolysis is one of the most used processes to convert biomass into energy due to its simplicity and low process temperature [151]. The pyrolysis of winery residues has been explored not only for the interesting HHV of the formed gas but also for the properties of the biochar formed (33–40%). The biochar formed during the pyrolysis has been indicated as an interesting fertilizer due to the concentrations of potassium (2.2–2.3%), phosphorus (0.1–0.6%), nitrogen (0.5–2.7%), and organic carbon (40.2–58.2%) [158,159,160]. Moreover, del Pozo et al. (2021) investigated the recovery of phenolic compounds through the pyrolysis process as an alternative to conventional extraction [161]. The study concluded that the tar from the pyrolysis is rich in these compounds due to the total decomposition of hemicellulose and cellulose and the partial decomposition of lignin. In fact, the phenolic content recovered in the non-aqueous phase of tar was higher than the content recovered through conventional extraction processes. Comparing the three thermal processes, pyrolysis is the process that operates at lower temperatures, followed by the gasification process and combustion. According to a recent techno-economic analysis of wine industry co-products [162], the results suggest that grape marc can be valorized through the pyrolysis process, while the drying of biomass represents an important role in terms of economic viability. Combustion is the best in terms of economic analysis, mainly because it is the most developed technology (with higher efficiencies and lower costs associated), while it seems the worst in terms of the environmental impact. Pyrolysis presented the best results in terms of the overall environmental impact (60% lower than combustion).

Overall, the published studies suggest that winery residues can be valorized through thermal conversion processes.

### 4.3. Soil Amendment Production

The composting process corresponds to biological aerobic degradation of the biodegradable organic matter until its stabilization, producing compost with further valorization as fertilizer (or soil amendment). Composting has been considered for the management and valorization of several bioresidues, and winery residues as well. Bustamante et al. (2009) studied the composting parameters that better describe the compost derived from winery residues [163]. The study evaluated four mixtures with different proportions of raw materials (M1: 54% grape stalks, 27% exhausted grape marc, 16% grape marc, 3% vinasses; M2: 56% grape stalks, 28% exhausted grape marc, 16% grape marc; M3: 80% exhausted grape marc, 20% cattle manure; M4: 79% exhausted grape marc, 21% poultry manure). Diaz et al. (2002), studied the composting of another four mixtures of winery residues (V0: 100% grape marc; V10: 90% grape marc, 10% vinasses; V20: 80% grape marc, 20% vinasses; V40: 60% grape marc, 40% vinasses) [164]. The product resulting from the composting of winery residues exhibits high organic matter content and macronutrients (K and P) as well as the absence of a phytotoxic effect [163,164]. The high concentration of phenolic compounds in these residues can present some challenges since they can hamper the activity of microorganisms and cause germination inhibition. However, Bustamante et al. (2009) observed a reduction in these molecules throughout the composting process [163]. Moreover, the ammonia formation and nitrogen losses increase with the increase in vinasses proportions in the composting piles [163,164]. Overall, the composting of winery residues resulted in a high-quality compost when moderate amounts of winery wastewater were used and the proportion between the different raw materials was optimized [163,164]. In particular, some properties reported in the compost demonstrated good quality: C/N between 10.5 and 32.8, N-NH_4_^+^ between 3.1 and 58.0 mg kg^−1^, N-NO_3_^−^ between 10.5 and 97.4 mg kg^−1^, P in the range 3.4–9.6 g kg^−1^, absence of E. coli and Salmonella spp, and metals in low concentrations (lower than regulatory limits) [163,164]. In this scope, it must be mentioned that the anaerobic digestate that remains after anaerobic digestion can be used as a composting substrate since it is a mixture of partially stabilized organic matter and is a source of carbon, nitrogen, and phosphorus. The direct application of the digestate into the soil is not recommended due to the possible presence of phytotoxic compounds, such as ammonia and volatile fatty acids [135]. The composting process has been proposed a post-treatment to stabilize the organic matter of the anaerobic digestate and achieve a good maturity degree [165]. However, due to the high-water content of the digestate, bulking agents are commonly needed to increase porosity and ensure efficient aeration. According to Bustamante et al. (2012), both vine shoot pruning and exhausted grape marc can be used as bulking agents [166]. In this situation, it is possible to achieve thermophilic conditions (temperature higher than 40 °C) inside the composting mixture, and consequently a reduction in the electric conductivity and nitrogen loss was observed. It should be stressed that the production of compost with winery residues may not only have a very relevant agronomic value for returning stable organic matter and nutrients to the soil (for example in vines) but also this approach may give a positive contribution to climate change (fixing carbon in soil). 

## 5. Integrated Biorefinery Approaches: Case Studies

**Figure 3:**
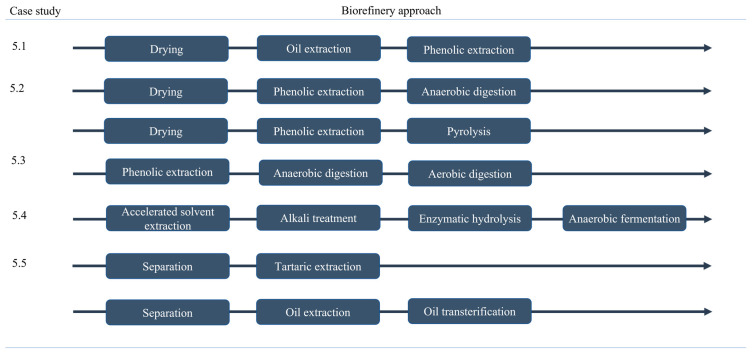
Strategies of biorefinery investigated and presented as case studies in Section 5.1, Section 5.2, Section 5.3, Section 5.4 and Section 5.5.

The biorefinery is the combination of different processes that allows for the maximum valorization of biomass into products and/or energy. Depending on the waste source and target products, various processes can be combined in a cascading approach, whereas it is not possible to indicate a particular combination that can be applied to all types of waste. Although winery residues have been referred to as a suitable and promising feedstock for integrated biorefinery approaches, there are a scarce number of studies that explore the integration of two or more processes using these types of residues. In this study, five specific case studies of an integrated biorefinery approach applied on winery residues were explored and summarized. Figure 3 summarizes the particular biorefinery approaches explored in Section 5.1, Section 5.2, Section 5.3, Section 5.4 and Section 5.5.

### 5.1. Grape Seed Oil Extraction Followed by Phenolic Compounds Extraction

A recent study conducted by Lucarini et al. (2018) explored the feasibility of recovering phenolic compounds after oil extraction from dried grape seeds [167]. According to the results, the mechanical extraction of oil resulted in a yield of 15%. After the oil extraction, the remaining seed residues were submitted to a hydroalcoholic extraction (ethanol:water 70:30, pH 2.5 by HCOOH addition) and an aqueous extraction. From the hydroalcoholic extraction, the authors manage to extract 43.88 mg of total phenolic compounds per g of extract. However, aqueous extraction resulted in lower phenolic content extracted. Overall, Lucarini et al. (2018) concluded that is possible to recover phenolic compounds after oil extraction from grape seeds. However, more studies and optimization of the processes are still needed [167].

### 5.2. Grape Pomace Phenolic Extraction Followed by Anaerobic Digestion or Pyrolysis

Almeida et al. (2021) evaluated the impact of phenolic compounds extraction in methane production through the anaerobic digestion of grape pomace [168]. The authors evaluated the performance of the biochemical methane potential assays before and after the extraction process with ethanol. The results showed that the grape pomace exhibited a production of 176 L of methane per kg of volatile solids prior to the phenolic extraction. The extraction of phenolic compounds using ethanol as solvent allowed for the recovery of 56 mg_GAE_ per g of grape pomace extract. After the extraction, the remaining grape pomace was submitted to a new biochemical methane potential test in which a production of 120 L of methane per kg_VS_ was obtained. Moreover, through statistical analysis, the authors concluded that the recovery of phenolic compounds based on ethanol extractions did not affect the methane potential of the grape pomace, making this residue an interesting substrate for this integrated approach. Moreover, Almeida et al. (2022) evaluated the impact of the phenolic compounds in the pyrolysis process, as well as the comparison between the energetic capacity of the gases produced during the anaerobic digestion and pyrolysis [169]. Once again, the recovery of phenolic compounds before pyrolysis did not affect negatively the process. According to Almeida et al. (2022), the pyrolysis plateau was reached at a temperature of 700 °C, where 20% gas, 40% tar, and 40% biochar yields were obtained [169]. When comparing the energy recovered through biogas and syngas formed by pyrolysis, the authors concluded that based only on the gaseous products, winery residues should be processed through anaerobic digestion.

### 5.3. Grape Pomace Phenolic Extraction Followed by Two-Stage Anaerobic Digestion and Aerobic Digestion

Martinez et al. (2016) studied a cascading biorefinery approach for the production of polyphenols, volatile fatty acids (VFAs), polyhydroxyalkanoates (PHA), and biomethane from red grape pomace [170]. The raw grape pomace was submitted to two different extraction processes, namely, the supercritical extraction using CO_2_ containing 10% of aqueous ethanol (57% *v*/*v*) and the conventional extraction using methanol as solvent. The different extractions resulted in similar phenolic content being extracted (about 180 mg_GAE_ per g of extract). The remaining grape pomace from the supercritical extraction step was then submitted to a batch anaerobic acidogenic wet process in order to produce a mixture of VFAs, namely, acetic acid (15.5 g L^−1^) and butyric acid (4.3 g L^−1^). According to the authors, the preliminary extraction of phenolic compounds did not affect the acidogenic phase of anaerobic digestion. Furthermore, the resulting VFA-rich mixture was used as a substrate for the biotechnological production of PHA, which can be used to produce biopolymers. The leftover solid fraction from the extraction and acidogenic step were digested under anaerobic conditions in order to produce biomethane. From this study, the authors confirmed the suitability of grape pomace for an integrated biorefinery approach.

### 5.4. Grape Pomace Extraction Followed by Enzymatic Hydrolysis and Anaerobic Fermentation

Jin et al. (2018) studied the cascading biorefinery approach for the production of oil, polyphenols and an acetone–butanol–ethanol mixture [132]. The powder of red grape pomace was firstly submitted to an accelerated solvent extraction process with hexane in order to obtain oil. The ASE of grape pomace resulted in a yield of 71.9 mg of oil per g of grape pomace. Moreover, the oil obtained presented a large quantity of unsaturated fatty acids mainly composed of linoleic acid. According to the authors, this oil has interesting properties to be further used in food, cosmetic and pharmaceutical industries. After the oil extraction, the remaining solid fraction was submitted to a new ASE process this time using a hydroalcoholic solution as solvent. The obtained extracts were found to be rich in phenolic compounds (73 m_GAE_ g^−1^, dry basis). In fact, when compared to the extracts obtained through UAE (using acetone + water + acetic acid as solvent), the ASE extracts exhibited 7 times the phenolic concentration as well as higher antioxidant activity. The oil-free phenolic-free grape pomace was then pre-treated with NaOH in order to remove the lignin and consequently increase its bio-accessibility. After the alkali treatment, the remaining solid fraction was processed through an enzymatic hydrolysis, which resulted in a production of 167 mg of sugars per g of pre-treated grape pomace. In fact, the quantity of sugars obtained was two times the concentration obtained using raw grape pomace. The hydrolyzed sugars obtained were subjected to an anaerobic fermentation where a mixture of acetone–butanol–ethanol was obtained. Overall, the authors concluded that it is feasible to valorize the grape pomace through an integrated biorefinery approach, where 1 kg of dried grape pomace can result in 71.9 g of crude oil, 322.8 g of phenolic extract and 20.7 g of acetone–butanol–ethanol mixture.

### 5.5. An Italian Case Study for Upgrading Wineries to Biorefinery and Circular Economy Framework

According to Ncube et al. (2021), this study is the first one that evaluated the design of a circular winery through a life cycle assessment (LCA) [171]. The authors studied a particular case of an Italian winemaking industry through the creation of two scenarios. The first scenario is the linear production (traditional winery), whereas the second scenario takes into account a circular process based on the reuse of the generated by-products. For this analysis, all stages from the cultivation to the valorization of wine lees, grape pomace, and prunings/stalks were considered. The LCA included three subsystems: agricultural phase, wine production phase, and bottling phase. Moreover, three additional scenarios were considered: improved agricultural phase (the diesel is replaced by biodiesel obtained from grape seeds extraction and the use of biofertilizers from exhausted grape pomace); improved vinification phase (the electricity is produced through steam obtained from vine prunings valorization); improved side production chain (the steam used in the distillation is substituted by a bio-steam recovered from vine prunings and grape stalks). The LCA results demonstrated that the circular production systems lead to a reduction in the negative impacts associated with the winemaking industry. These improvements were particularly demonstrated in the global warming, freshwater eutrophication, and mineral resource scarcity impact categories. Moreover, the study confirmed that there is a need for the development of new technologies and processes as well as the creation of new and more competitive markets to achieve the UN Sustainable Development Goals and climate action. Based on this study, it is advisable to shift from a linear winemaking process to a circular system based on a biorefinery approach, leading to a more sustainable and eco-friendly industry.

## 6. Conclusions and Forthcoming Developments

The winery industry is a very important sector of activity in many countries worldwide, but the residues generated can have negative environmental impacts if not properly managed. Based on the current knowledge about the value of these residues, efficient and eco-friendly strategies are necessary to extract value using these residues as valuable raw materials in multiple processes. Taking into account the composition of these residues, several investigations have been conducted to demonstrate the feasibility of bioactive compound extraction and energy recovery. 

The published studies support the suitability and economic value when a cascade biorefinery approach is applied, instead of only one process at a time. Clearly, real integrated biorefinery approaches are currently poorly explored, and mainly at the laboratory level. Therefore, it seems urgent to scale-up the proven strategies and evaluate the associated costs. Moreover, the literature shows a gap in approaches based on life-cycle assessment (LCA) and technical-economical evaluations. In this scope, it seems valuable to mention the study performed by Ncube et al. (2021), who performed an LCA regarding the design of a circular winery instead of a linear traditional winemaking process [171]. The main conclusion was that the shift from a linear to a circular system can reduce some of the negative impacts of winemaking, particularly global warming, freshwater eutrophication, and mineral resource scarcity. Moreover, this study highlighted that those innovative processes and technologies will be valuable to shift from linear systems to biorefinery approaches.

Overall, in the future, winery residues must be taken into account to be used as feedstock for integrated biorefineries due to the presence of high-value organic compounds, as well as for energy production or fertilizers. 

## Figures and Tables

**Table 1:** General characterization of winery wastewater.

Parameter	Value	Parameter	Value
pH	3.10–12.90	P (mg L^−1^)	3.30–188.3
Electrical conductivity (dS m^−1^)	0.14–72.0	Fe (mg L^−1^)	1.00–77.0
Total solids (g L^−1^)	0.19–79.6	Mg (mg L^−1^)	1.96–1170
Volatile solids (g L^−1^)	0.66–54.9	Ca (mg L^−1^)	12.00–2203
Chemical oxygen demand (g L^−1^)	0.34–296	Mn (mg L^−1^)	200.00–1740
Biochemical oxygen demand (g L^−1^)	0.004–41.0	Cu (mg L^−1^)	0.05–3260
Total organic carbon (g L^−1^)	0.11–20.9	Zn (mg L^−1^)	12.00–1400
Total phenolic content (mg L^−1^)	29.00–1450.	Ni (mg L^−1^)	500–650
Total carbohydrates (g L^−1^)	1.56–1.56		
Total lipids (g L^−1^)	0.25–0.25		
Total proteins ((g L^−1^)	0.01–2.75		
Total Kjeldahl nitrogen (g L^−1^)	0.03–0.07		

References: [3,29,30,31,32,33,34].

**Table 2:** General composition of grape seeds.

Parameter	Value
pH	4.6
Moisture (%)	5.5–34.8
Organic matter (%, db)	94.2–96.2
Ash (%, db)	2.1–8.3
Klason lignin (%, db)	50.7 ^a^
Total carbohydrates (%, db)	64.7–72.1
Diatery fibers (%, db)	35.3
Lipids (%, db)	7.2–24.8
Proteins (%, db)	5.0–19.0

^a^ after defatting step. References: [35,36,37,38,39,40].

**Table 3:** General composition of grape stalks.

Parameter	Value	Parameter	Value
Moisture (%)	56.8–76.7	Proteins (%, db)	4.9–11.2
Organic matter (%, db)	89.0–94.5	K (g kg^−1^, db)	9.00–28.73
Ash (%, db)	3.9–11.2	Ca (g kg^−1^, db)	1.50–5.97
Klason lignin (%, db)	4.6–47.3	Mg (g kg^−1^, db)	0.20–2.63
Hemicelluloses (%, db)	13.9–24.5	Zn (g kg^−1^, db)	0.01–0.10
Cellulose (%, db)	30.3	Na (g kg^−1^, db)	0.10–0.32
Total carbohydrates (%, db)	14.0–27.6	Fe (g kg^−1^, db)	0.07–0.25
Soluble carbohydrates (%, db)	5.1–12.4	Cu (g kg^−1^, db)	0.04–0.05
Diatery fibers (%, db)	1.0–77.2	Mn (g kg^−1^, db)	0.09–0.17
Lipids (%, db)	0.9–3.4	Mg (g kg^−1^, db)	2.33–2.63

References: [4,9,35,50,54,55].

**Table 4:** General composition of grape skins.

Parameter	Value	Parameter	Value
Moisture (%)	5.6	Ca (g kg^−1^, db)	3.93–4.26
Organic matter (%, db)	81.7–98.0	Fe (g kg^−1^, db)	0.17–0.72
Ash (%, db)	2.0–18.3	K (g kg^−1^, db)	17.92–24.68
Klason lignin (%, db)	22.4–22.5	Mg (g kg^−1^, db)	0.40–0.51
Hemicelluloses (%, db)	3.6–12.5	Na (g kg^−1^, db)	0.17–0.27
Cellulose (%, db)	20.8–25.9	Zn (g kg^−1^, db)	0.01–0.02
Diatery fibers (%, db)	19.3		
Proteins (%, db)	5.0–19.0		

References: [35,56,57,58].

**Table 5:** General composition of grape pomace.

Parameter	Value	Parameter	Value
Moisture (%)	50.0–72.0	Lipids (%, db)	19.9–74.5
Organic matter (%, db)	50.2–72.2	Proteins (%, db)	2.7–12.2
Ash (%, db)	82.7–95.90	K (g kg^−1^, db)	11.80–37.90
Klason lignin (%, db)	32.5–56.7	Ca (g kg-1, db)	5.40–20.60
Hemicelluloses (%, db)	6.9–8.0	Mg (g kg^−1^, db)	0.70–2.20
Cellulose (%, db)	17.5–25.3	Zn (g kg^−1^, db)	0.01–0.04
Total carbohydrates (%, db)	18.2	Fe (g kg^−1^, db)	0.54–0.28
Soluble carbohydrates (%, db)	29.0-31.4	Cu (g kg^−1^, db)	0.01–0.28
Diatery fibers (%, db)	2.0–17.2	Mn (g kg^−1^, db)	0.0002–0.10

References: [35,36,50,60,65,66,67,68].

**Table 6:** Summary of solid–liquid extraction for the recovery of phenolic compounds from solid winery residues.

Operational Conditions	Sample	TPh (mg_GAE_ g_db_^−1^)	Reference
Solvent: Ethanol:Water (49:51); t: 5 min; T: 50 °C; L/S: 16.5	Skins	13.29	[84]
Solvent: Ethanol:Water (70:30); t: 300 min; T: 60 °C; L/S: 4	Pomace	19.17	[85]
Solvent: Ethanol:Water (50:50); t: 30 min; T: 30 °C; L/S: 10	Skins	48.6	[86]
Solvent: Methanol:HCl (99.9:0.1); t: 60 min; T: 4 °C; L/S: 50	Pomace	74.75	[48]
Solvent: Acetone:Water (70:30); t: 120 min; T: 60 °C; L/S: 20	Pomace	17	[87]
Solvent: Methanol:Acetone:Water (60:30:10) with 0.1% HCl; t: 10 min; T: nd; L/S: nd	Pomace	54.02	[88]
	Stalks	57.98	
	Seeds	103.3	
	Skins	36.25	
Solvent: Methanol; t: 30 min; T: nd; L/S: 10	Pomace	5.33	[89]
Solvent: Ethanol:Water (80:20) with 0.5% HCl (0.1 N); t: nd; T: nd; L/S: 30	Skins and Seeds	41.9	[90]
	Seeds	85.8	
	Skins	33.3	
Solvent: Citric Acid (2%): Water (3:1); t: 1440 min; T: RT; L/S: nd	Pomace	3	[91]
Solvent: Water; t: 45 min; T: 45 °C; L/S: 20	Stalks	27.09	[92]
Solvent: Ethanol; t: 1140 min; T: 25 °C; L/S: 5	Pomace	7.87	[93]
Solvent: Ethyl acetate:Methanol:Water (60:30:10); t: 480 min; T: 60 °C; L/S: 2	Skins	45.44	[94]
Solvent: Acetone:Water:Acetic acid (90:9.5:0.5); t: 480 min; T: 60 °C; L/S: 2	Seeds	667.9	
Solvent: Ethanol:Water (50:50); t: 120 min; T: 60 °C; L/S: 20	Pomace	18	[95]
Solvent: Choline chloride:Malic acid:Water (25:25:25); t: 180 min; T: RT; L/S: 60	Wine lees	5.5 ^a^	[96]

^a^—mg anthocyanins g_db_^−1^; L/S—liquid–solid ratio; T—temperature: t—time; TPh—total phenolic content expressed in db; GAE—gallic acid equivalents; db—dry basis; nd—not defined; RT—room temperature.

**Table 7:** Summary of ultrasound-assisted extraction for the recovery of phenolic compounds from solid winery residues.

Operational Conditions	Sample	TPh (mg_GAE_ g_db_^−1^)	Reference
Solvent: Ethanol:Water (50:50); t: 9 min; T: 28 °C; Power: 20 kHz, 1000 W; L/S: 10	Skins	80	[86]
Solvent: Citric Acid (2%):Water (3:1); t: 15 min; T: 88.1 °C; Power: 450 W; L/S: nd	Pomace	4.5	[91]
Solvent: Water; t: 45 min; T: 45 °C; Power: 25 kHz, 200 W; L/S: 20	Stalks	31.89	[93]
Solvent: Acetone:Water (50:50); t: 15 min + 30 min; T: RT; Power: nd; L/S: 10	Seeds	41	[97]
Solvent: Ethanol:Water (47:53); t: 20 min T: 56 °C; Power: 20 kHz; 130 W; L/S: 8	Pomace	48.76	[98]
Solvent: Ethanol; t: 15 min; T: 80 °C; Power: 35 kHz; L/S: nd	Pomace	6	[99]
Solvent: Ethanol:Water (50:50); t: 15 min T: nd; Power: 40 kHz; L/S: 200	Pomace	26.21	[100]
Solvent: 2-propanol:Water (50:50); t: 29.6 min; T: 50 °C; Power: 25 kHz; L/S: 10	Pomace	62.49	[101]
Solvent: Ethanol:Water (50:50) + 2.4% citric acid; t: 3 min; T: 26 °C; Power: 20 kHz, 2600 W;L/S: 13	Skins	117.3	[102]
Solvent: Ethanol:Water (50:50); t: 25 min; T: 50 °C; Power: 40 kHz, 250 W; L/S: 50	Seeds	26.6	[103]
Solvent: Methanol:Ethanol:Water (50:25:25); t: 20 min; T: nd; Power: nd; L/S: 10	Skins and Seeds	61.8	[104]
Solvent: Methanol; t: 15; T: nd; Power: nd; L/S: 10	PomaceSeeds	41 a	[105]
Solvent: Choline chloride:Malic acid:Water (25:25:25); t: 30.6 min; T: RT; Power: 37 kHz, 341.5 W;L/S: 10	Wine lees	6.6 ^a^	[96]

^a^—mg anthocyanins g_db_^−1^; L/S—liquid–solid ratio; T—temperature: t—time; db—dry basis; nd—not defined; RT—room temperature; a (mg_GAE_ L-1)/g_sample_, db.

**Table 8:** Summary of microwave-assisted extraction for the recovery of phenolic compounds from winery residues.

Operational Conditions	Sample	TPh (mg_GAE_ g_db_^−1^)	Reference
Solvent: Ethanol:Water (50:50); t: 30 min; T: nd; Power: 3458 MHz; 1000 W; L/S: 10	Skins	104	[86]
Solvent: Ethanol:Water (42:58); t: 5 min T: nd; Power: 408 W; L/S: 24			
Solvent: Citric Acid (2%):Water (3:1); t: 10 min; T: 92.5 °C; Power: 1000 W; L/S: nd	Pomace	7	[91]
Solvent: Methanol:Water (60:40); t: 16 min; T: 60 °C; Power: 2.45 GHz; L/S: 50	Skin	22.16	[109]
Solvent: Ethanol:Water (30:70); t: 15 min; T: 24 °C; Power: 93 W; L/S: 6.7	Pomace	10.2	[110]

L/S—liquid–solid ratio; T—temperature: t—time; db—dry basis; nd—not defined; RT—room temperature.

**Table 9:** Summary of accelerated-solvent extraction for the recovery of phenolic compounds from winery residues.

Operational Conditions	Sample	TPh (mg_GAE_ g_db_^−1^)	Reference
Solvent: Ethanol:Water (48.81:51.19); t: 14.82 min; T: 50.79 °C; Pressure: 10.1 MPa; S/F: nd	Skins	15.24	[84]
Solvent: Water; t: 130 min; T: 140 °C; Pressure: 8 MPa; S/F: nd	Pomace	32.49	[92]
Solvent: Ethanol:Water (50:50); t: 6 min T: 120 °C; Pressure: 10.3 MPa; S/F: nd	Stalks	57.1	[111]
Solvent: Ethanol:Water (50:50), pH 2; t: 40 min; T: 85 °C; Pressure: nd; S/F: 253	Skins and seeds	76.38	[112]

L/S—liquid–solid ratio; T—temperature: t—time; db—dry basis; nd—not defined; RT—room temperature.

**Table 10:** Summary of supercritical fluid extraction for the recovery of phenolic compounds from winery residues.

Operational Conditions	Sample	TPh (mg_GAE_ g_db_^−1^)	Reference
Solvent: CO_2_; Flowrate: nd; t: nd; T: 40 °C; Pressure: 30 MPa	Pomace	18	[95]
Solvent: CO_2_ + Ethanol (20%); Flowrate: 2 mL min^-1^; + 0.4 mL min-1; t: nd; T: 60 °C; Pressure: 25 MPa	Pomace Skins Seeds	0.570	[113]
		0.603	
		0.336	
Solvent: CO_2_ + Ethanol (15%); Flowrate: 6 kg h^-1^; t: 780 min; T: 40 °C; Pressure: 0.8 MPa	Pomace	71.32	[114]
Solvent: CO_2_ + Ethanol (5%); Flowrate: 2 mL min^-1^; t: 30 min; T: 46 °C; Pressure: 16.7 MPa	Seeds	85	[115]

L/S—liquid–solid ratio; T—temperature: t—time; db—dry basis; nd—not defined; RT—room temperature.

**Table 11:** Summary of high-voltage discharge extraction for the recovery of phenolic compounds from winery residues.

Operational Conditions	Sample	TPh (mg_GAE_ g_db_^−1^)	Reference
Solvent: Ethanol:Water (50:50), 1% HCl; Energy input: 22.27 kJ kg^−1^; t: 15 min; T: 25 °C; L/S: nd	Pomace	30	[99]
Solvent: Ethanol:Water (50:50), pH 5.5; Energy input: 188 kJ kg^−1^; t: nd T: 20 °C; L/S: 15	Stalks	78.8	[118]
Solvent: Water; Energy input: 53 kJ kg^−1^; t: nd; T: 20 °C; L/S: 5	Seeds	25	[120]
Solvent: Water; Energy input: 213 kJ kg^−1^; t: nd; T: 20 °C; L/S: 5	Stalks	1.85	
Solvent: Ethanol:Water (30:70); Energy input: 80 kJ kg^−1^; t: 60 min; T: 20 °C; L/S: 2	Pomace	28	[119]

L/S—liquid–solid ratio; T—temperature: t—time; db—dry basis; nd—not defined; RT—room temperature.

**Table 12:** Operational parameters, advantages and disadvantages [122] of each extraction method.

Parameter	SLE	UAE	MAE	ASE	SFE	HVED
t (min)	10–1440	9–45	5–30	6–130	30–780	15–60
T (°C)	4–60	26–80	24–93	51–140	40–60	20–25
Pressure (MPa)	atm	atm	atm	8–10	0.8–30	atm
L/S (mL g^−1^)	2–50	8–200	7–50	nd	nd	2–15
Advantages	Easy operation; widely used	Eco friendly; can be used for themo-sensible compounds; reduced extraction time	Reduced solvent usage; reduced extraction time; high extraction yield	Low solvent consuption; low extraction time	Eco friendly; can be used for themo-sensible compounds	Reduced extraction time, low solvente consuption,
Disadvantages	Impurities; analytical errors	Not uniform energy distribution; decline power with time	High capital costs	Not suitable for themo-sensible compounds	High capital costs; high pressure requirments	Low selectivity; high energy consuption

**Table 15:** Characteristics of bioethanol, bio-oil and biodiesel produced from winery residues.

Product	Parameter	Value	Reference
Bioethanol	Yeast culture	*Saccharomyces cerevisiae* 1072	[58]
	Agitation (rpm)	180	[58]
	Temperature ( °C)	28	[58]
	Sample (% *v*/*v*)	70	[58]
	Inoculum (% *v*/*v*)	20	[58]
	Supplementary medium (% *v*/*v*)	10	[58]
	Yield (mL kg^−1^)	310–400	[58,64]
Bio-oil	Yield (%)	6.4–7.2	[143,144]
	Unsaturated fatty acids (%)	84.6–89.0	[143,144,145,146]
	Saturated fatty acids (%)	11.0–15.4	[143,145,146]
Bio-diesel (FAEE)	Density, 15 °C (kg m^-3^)	879.5–882.4	[143,145]
	Kinematic viscosity, 40 °C (mm2 s^−1^)	4.1–4.7	[143,145,146]
	HHV (MJ kg^−1^)	40.0	[143]
	LHV (MJ kg^−1^)	37.5–37.6	[143,145]

FAEE—fatty acid ethyl esters; HHV—higher heating value; LHV—lower heating value; nd—not defined.

## Data Availability

Not applicable.
